# A machine-learning approach to discerning prevalence and causes of myopia among elementary students in Hubei

**DOI:** 10.1007/s10792-022-02279-5

**Published:** 2022-04-07

**Authors:** Yuyang Tu, Xuemin Hu, Caiqiong Zeng, Meihong Ye, Peng Zhang, Xiaoqin Jin, Jianwei Zhang, Lianhong Zhou

**Affiliations:** 1 Department of Informatics, University of Hamburg, Hamburg, Germany; 2 Department of Ophthalmology, Renmin Hospital of Wuhan University, Wuhan, 430060 Hubei Province China; 3 Department of Ophthalmology, Ezhou Central Hospital, Ezhou, Hubei Province China; 4 Wuhan Eyegood Ophthalmic Hospital, Wuhan, Hubei Province China

**Keywords:** Hubei Province, Elementary school students, Myopia, Machine-learning, Influencing factors

## Abstract

**Objective:**

Our aim is to establish a machine-learning model that will enable us to investigate the key factors influencing the prevalence of myopia in students.

**Methods:**

We performed a cross-sectional study that included 16,653 students from grades 1–3 across 17 cities in Hubei Province. We used questionnaires to discern levels of participation in potential factors contributing to the development of myopia. The relative importance of potential contributors was ranked using machine-learning methods. The students’ visual acuity (VA) was measured and those with logMAR VA of > 0.0 underwent a autorefraction test to determine students’ refraction status.

**Results:**

The prevalence of myopia in grades 1, 2, and 3 was 14.70%, 20.54% and 28.93%, respectively. Myopia rates among primary school students in provincial capital city (32.35%) were higher than those in other urban (23.03%) and rural (14.82%) areas. Children with non-myopic parents, only one myopic parent, or both parents having myopia exhibited myopic rates of 16.36%, 25.18%, and 41.37%, respectively. Myopia prevalence was higher in the students who continued to use their eyes at close range for a long time and lower in those engaged longer in outdoor activities. The machine-learning model determined that the top three contributing factors were the students’ age (0.36), followed by place of residence (0.34), starting age of education (0.21).

**Conclusion:**

The overall prevalence of myopia was 21.52%. Children’s age and place of residence were the important influencing factors, but genetics and environmental were also played key roles in myopia development.

## Introduction

The increasing prevalence of myopia is becoming a significant public health problem. Holden et al. [1] predicted that by 2050, 49.8% of the global population will suffer from myopia. High myopia can cause pathological changes in the retina, choroid, vitreous, leading to serious complications such as retinal detachment and neovascularization, macular hemorrhaging, and macular degeneration [2]. The higher the degree of myopia, the greater the possibility of complications (blindness can result in severe cases). The Beijing Eye Study found that pathological myopia (PM) was the cause of blindness in 7.7% (1 of 13 individuals) and low vision in 32.7%(16 of 49 individuals) of Chinese individuals [3].

The high incidence of myopia in children and adolescents is particularly concerning. A recent report from the National Health Commission of China revealed that 53.6% of Chinese children and adolescents nationwide in 2018 exhibited myopia. Across elementary, junior high, and high school students, these proportions were 36.0%, 71.6% and 81.0%, respectively. Myopia affects the growth and development of children and imposes a potential economic burden on the family and society. It has been calculated that the global potential economic loss related to myopia was approximately US$244 billion in 2015 alone, and East Asia, in particular, suffered the immense economic loss [4].

Myopia develops as a result of multiple factors and once it occurs, the condition is irreversible. During the growth and development period, myopia will increase yearly. The younger a patient is when first diagnosing myopia, the higher the probability of developing high myopia later in life [5]. As such, investing in the prevention and control of school-age children’s myopia is of utmost importance. A survey conducted by the National Health Commission of China in 2018 showed that the prevalence of myopia in elementary school students increased substantially from 15.7% in grade 1 to 59% in grade 6, suggesting that elementary school is the key stage to focus on for China’s prevention and control of myopia.

As “Big Data” becomes increasingly available, data mining and machine-learning technologies are rapidly improving. “Big data” refers to a data set with huge volume, various types and complex structure, which is difficult to be effectively analyzed and utilized by traditional data processing methods. Big data have 4 V characteristics: volume (large data scale); Variety (multiple data sources, multiple data types and strong correlation between data); Velocity (fast data growth and processing speed); Value (the hidden value of big data is huge) [6]. Here, we leverage Big Data-based machine-learning methods to analyze the influence of factors predicted to affect myopia’s development among elementary school students. We first conducted a survey of myopia-related factors in elementary school students from 17 cities in Hubei Province, China, and conducted visual acuity and refractive status examinations for students who returned valid questionnaires. Subsequently, we built machine-learning models to analyze various effects of myopia’s prevalence and identify important influencing and risk factors. Ultimately, this study was to supplement the blank of large-scale epidemiological survey data of myopia in Central China, and use a new method (machine learning model) to mine the influencing factors of myopia from big data, so as to provide a scientific and reasonable basis for the prevention and treatment of children's myopia.

## Subject and method

### Subject of investigation

We performed a cross-sectional investigative study. The prevalence of myopia in this age group across China’s inland area was reported to be 26.5% [7], the confidence is 95%, *t*_0.05_ = 1.96, the allowable error (*d*) is 5%, *N* = *t*_0.05_^2^*p*(1 − *p*)/*d*^2^ = 1.98^2^ × 0.265 × (1 − 0.265)/0.05^2^ = 306. Allowing for non-response and lost questionnaires, we calculated that each survey effort should include > 500 students. Using a cluster stratified sampling method, we selected Wuhan City, Jingmen City, Shiyan City, Tianmen City, Huangshi City, Jingzhou City, Qianjiang City, Xiantao City, Suizhou City, Xianning City, and Xiaogan City in total 11 cities in Hubei Province as urban survey points, and Songbai Town in Shennongjia Forest District, Qichun prefectural in Huanggang City, Badong County in Enshi Autonomous Prefecture, Miaoeling Village in Ezhou City, Nanzhang prefecture in Xiangfan City, and Wujia Township in Yichang City 6 district in total were selected as rural survey points. Two schools were then selected from each survey point. In each school, we conducted random sampling based on class to select a total of 16,653 elementary school students (6–10 years old), including 11,365 urban students and 5288 rural students. Ethics approval was obtained from the Clinical Research Ethics Committee of Renmin Hospital of Wuhan University (WDRY 2020-K211) and all research was carried out according to the tenets of the Declaration of Helsinki involving human participants. Before participating in the survey, parents of all participating students and the students themselves provided written informed consent. In total, 15,013 students completed the questionnaire and the visual acuity and refractive examination (overall response rate: 90.15%). We received responses from 10,214 students from urban areas and 4799 students from rural areas; of these, 4987 were in the first grade, 4796 were in the second grade, and 5230 were in the third grade. 8273 of the participants were boys and 6740 were girls. Children with glaucoma, corneal disease, medium refractive opacity, fundus lesions, strabismus or other eye diseases which affect visual acuity, those with a history of eye surgery, and those receiving atropine eye drops and orthokeratology were excluded from the survey.

### Examination items

1. *Questionnaire survey*: The questionnaire was completed by parents and students. The questionnaire included students’ physiological factors (e.g., gender, age, height, and weight), birth history (e.g., delivery mode, whether preterm birth), information about their parents (e.g., whether myopic, education level), near work behavior (e.g., time for outdoor activities, continuous reading and writing time, screen time, and extracurricular training time), dietary habits, and asked parents to provide information on their recognition and awareness of myopia. The refractive status of each participating students’ parents was recorded and divided into categories of mild myopia (SE: − 3.00 D ~ 0.50 D), moderate myopia (SE: − 6.00 D ~  − 3.00 D), and severe myopia (SE <  − 6.00 D).
2. *Visual acuity (VA)*: The students who have returned the qualified questionnaire were checked for visual acuity. Acuity was measured with a unified standard logarithmic visual acuity chart (National Standard of People's Republic of China, GB11533-2011) at an inspection distance of 5 m. The VA was recorded in a decimal scale and converted to logMAR for analysis.
3. *Refractive examination*: Students with a logMAR VA of > 0.0 underwent cycloplegic autorefraction (TOPCON RM-8800), in which 1% cyclopentolate eye drops were administered to their eyes every five minutes for a total of three times. Forty minutes after the last drop, all participants underwent autorefraction, which is expressed in spherical equivalent (SE). A student was diagnosed with myopia if at least one eye has a poor VA (logMAR VA of > 0.0) and an SE of <  − 0.50 D.
4. *Eye routine examination*: As part of the examination, we performed tonometer, light microscopic, ophthalmoscopic, and eye movements examinations.

### Quality control

1. *Staff training*: We trained all medical staff involved in the subject, the standardized examination methods, and judgment criteria. We explained the contents of the questionnaire to ensure that the guardians and students fill in the questionnaire correctly.
2. *On-site supervision*: The personnel in charge of the research team were present on-site to ensure the correct implementation of the standard workflow.
3. *Instrument maintenance*: The instrument manufacturers and the ophthalmology professional personnel calibrated and inspected instrumentation used to minimize deviation in inspection results due to machine error.
4. *Data management*: After each on-site inspection, the on-site supervisor and data administrator checked the original data to uncover and correct missing or wrong items. All data were entered twice and archived after verification. For inconsistent items, the original data were again verified to ensure that the input data were accurate and reliable.
5. *Supplementary inspection*: If the inspection was not completed or the questionnaire was disqualified due to sick leave or other reasons, the school contacted the students and parents to ensure the data’s completeness and accuracy.

### Data processing

We obtained 15,013 valid questionnaires with clinical refraction data. Values are assigned to myopia-related influencing factors (Table 1). We used SPSS 23.0 Chi-square test to evaluate the difference in myopia prevalence among different grades, genders, regions, genetics, and environmental factors. Results were considered significant at *P* < 0.05. Taking 70% of the data as the training set, tenfold cross-validation was applied for internal validation; the remaining 30% were used for external validation. A total of 31 factors among six aspects related to myopia were chosen as input variables. The response variable used as whether the student displayed myopia.

**Table 1:** Assignment of factors related to myopia in primary school students of grade 1–3 in Hubei Province, China

*Target*	0	1	2	3
If the child was myopic	Non-myopic	Myopic		
*Factor*				
*Student general status*				
Grade	Grade 1	Grade 2	Grade 3	
Gender	Male	Female		
*Birth history*				
Way of birth	Cesarean section	Normal delivery		
Premature baby	No	Yes		
History of oxygen inhalation	Haven’t	Have		
History of ROP	Haven’t	Have		
*Parental status*				
Degree of myopia	No	Mild	Moderate	High
Education level	Low	Secondary	Higher	
Occupational category	Knowledge worker	Knowledge worker + Part of manual workers	Manual workers	
*Near work behavior*				
Outdoor activity time	< 0.5 h	0.5-1 h	1-2 h	> 2 h
Reading and writing time	< 0.5 h	0.5-1 h	1–1.5 h	> 1.5 h
If take brake after 0.5 h near work	No	Yes		
Most frequently used electrical device	TV	PC	Smart phone	Tablet
Electronic devices usage time	< 0.5 h	0.5-1 h	1–1.5 h	> 1.5 h
Extracurricular training time	No	< 1 h	1-2 h	> 2 h
*Dietary habits*				
Binge Eating	No	Yes		
Like sweets and carbonated drinks	Never	Sometimes	Frequently	
*Parental cognition*				
If take child to the hospital regularly for visual inspection	No	Yes		
Whether the child has eye abnormalities	No	Yes		
Parents’ knowledge about vision care	Don’t know	A little bit	Basic	Very well
Supervision of children’s vision protection	Never	Sometimes	Frequently	
If the child is short-sighted, whether to let the child wear glasses	No	Yes		
Parental awareness about the degree of hazards of myopia	Light	Moderate	Heavy	Severe

### Myopia prediction model

**Fig. 1:**
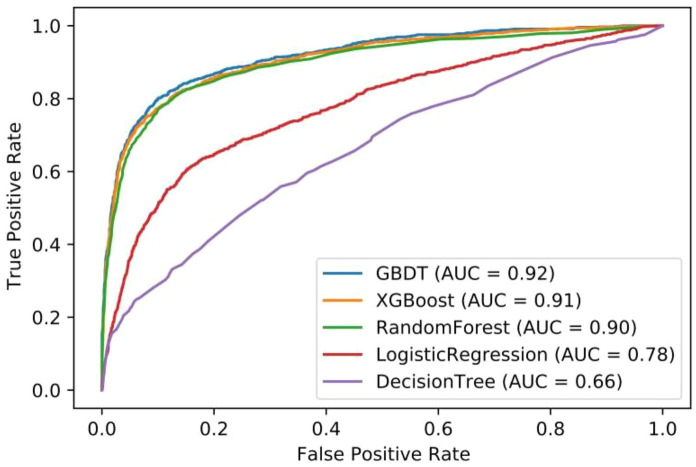
ROC curve of five selected machine-learning algorithms

The problem was formulated as a classification task. Five machine-learning models (Gradient Boosting Decision Tree (GBDT), Logistic Regression (LR), Decision tree (DT), Random Forest (RF), and eXtreme Gradient Boosting (XGBoost)) were developed to predict whether a student had myopia. We used Bayesian Optimization to tune hyperparameters for each model. AUC score was used as the evaluation metric to compare prediction performance across models automatically. The ROC curve was generated to show each models’ performance on the test dataset (Fig. 1). Ultimately, we chose the best performing model (GBDT) for subsequent feature importance analysis. Validation on both internal and external data indicated that the model has a good generalization to unseen data. As a tree-based model, GBDT allows for high-power statistical feature importance analysis [8]. We used the SHAP python package to calculate the Shapley values of each influencing factor in the GBDT model [9], representing a measure of impact (positive or negative) of the observed value’s contribution to each prediction. Feature importance from the GBDT model was calculated and ranked using the mean absolute Shapley values for each variable across all measurements. The higher the overall feature importance, the more impact the feature had on the occurrence of myopia.

## Result

### Status of myopia in elementary school students (6–10 years old) in Hubei Province

Across the 15,013 elementary school students, overall prevalence of myopia was 21.52% (14.70% in first grade, 20.54% in second grade and 28.93% in third grade). Myopia prevalence increased with grade level (*χ*^2^ = 310.140, *P* < 0.001) and was higher in girls than in boys (23.64% vs. 19.80%, *χ*^2^ = 32.353, *P* < 0.001).

Myopia prevalence in children with non-myopic parents, one myopic parent, or both myopic parents was 16.36%, 25.18%, and 41.37%, respectively; prevalence among children with both myopic parents was significantly higher than either other cases (*χ*^2^ = 588.537, *P* < 0.001). Myopia prevalence in students whose father had no myopia, mild, moderate, and high myopia was 17.97%, 27.34%, 37.88%, and 43.29%, respectively (*χ*^2^ = 438.622, *P* < 0.001) and prevalence for those whose mother had no myopia, mild, moderate, and high myopia was 17.71%, 30.20%, 34.85%, and 43.45%, respectively (*χ*^2^ = 429.564, *P* < 0.001). 65.15% (2391/3670) and 58.34% (2131/3653) of parents with myopia had higher education, while 35.80% (4061/11343) and 32.15% (3652/11360) had higher education among those with no myopia.

The prevalence of myopia is higher in students who continuously read and write, receive extracurricular education for long hours, and do not take a break after doing near work for half an hour. The prevalence of myopia is lower in students who spend long periods of time outdoors (*χ*^2^ = 35.684, 37.300, 50.030, all *P* < 0.001). Prevalence of myopia in students who most primarily utilize smartphones (25.01%) is significantly higher than that in students preferentially using computers (22.96%), tablets (21.72%) and TV (19.55%) (*χ*^2^ = 52.039, *P* < 0.001; Tables 2, 3).

**Table 2:** The basic situation of myopia among elementary school student of grade 1–3 in Hubei Province

Group	Total	Myopia	Ratio (%)	*X*^2^	*P*
*Grade*					
Grade1	4987	733	14.70	310.140	< 0.001
Grade2	4796	985	20.54		
Grade3	5230	1513	28.93		
*Gender*					
Male	8273	1638	19.80	32.353	< 0.001
Female	6740	1593	23.64		
*Parents’ status of myopia*					
None	9445	1545	16.36	588.537	< 0.001
One of pair	3813	960	25.18		
Both	1755	726	41.37		
*Father’s degree of myopia*					
No myopia	11,360	2041	17.97	438.622	< 0.001
Mild myopia	2041	558	27.34		
Moderate myopia	1217	461	37.88		
High myopia	395	171	43.29		
*Mother’s degree of myopia*					
No myopia	11,343	2009	17.71	429.564	< 0.001
Mild myopia	2030	613	30.20		
Moderate myopia	1205	420	34.85		
High myopia	435	189	43.45

**Table 3:** Eye use behavior of elementary school student in grades 1–3 in Hubei Province

Group	Total	Myopia	Ratio (%)	*X*^2^	*P*
*Outdoor activity time*					
< 0.5 h	1224	338	27.61	70.814	< 0.001
0.5-1 h	4946	1162	23.49		
1-2 h	5812	1203	20.70		
> 2 h	3031	528	17.42		
*Reading and writing time*					
< 0.5 h	2732	539	19.73	35.684	< 0.001
0.5-1 h	6775	1381	20.38		
1–1.5 h	3733	848	22.72		
> 1.5 h	1773	463	26.11		
*If take brake*					
No	4697	1176	25.04	50.030	< 0.001
Yes	10,316	2055	19.92		
*Extra-curricular training time*					
No	6097	1217	19.96	37.300	< 0.001
0.5-1 h	5229	1154	22.07		
1–1.5 h	2368	591	24.96		
> 1.5 h	1517	428	28.21		
*Most commonly used electronic devices*					
Smart phone	4174	1044	25.01	52.039	< 0.001
PC	1633	375	22.96		
Tablet	571	124	21.72		
TV	8635	1688	19.55		

### Analysis of the risk factors of myopia

**Fig. 2:**
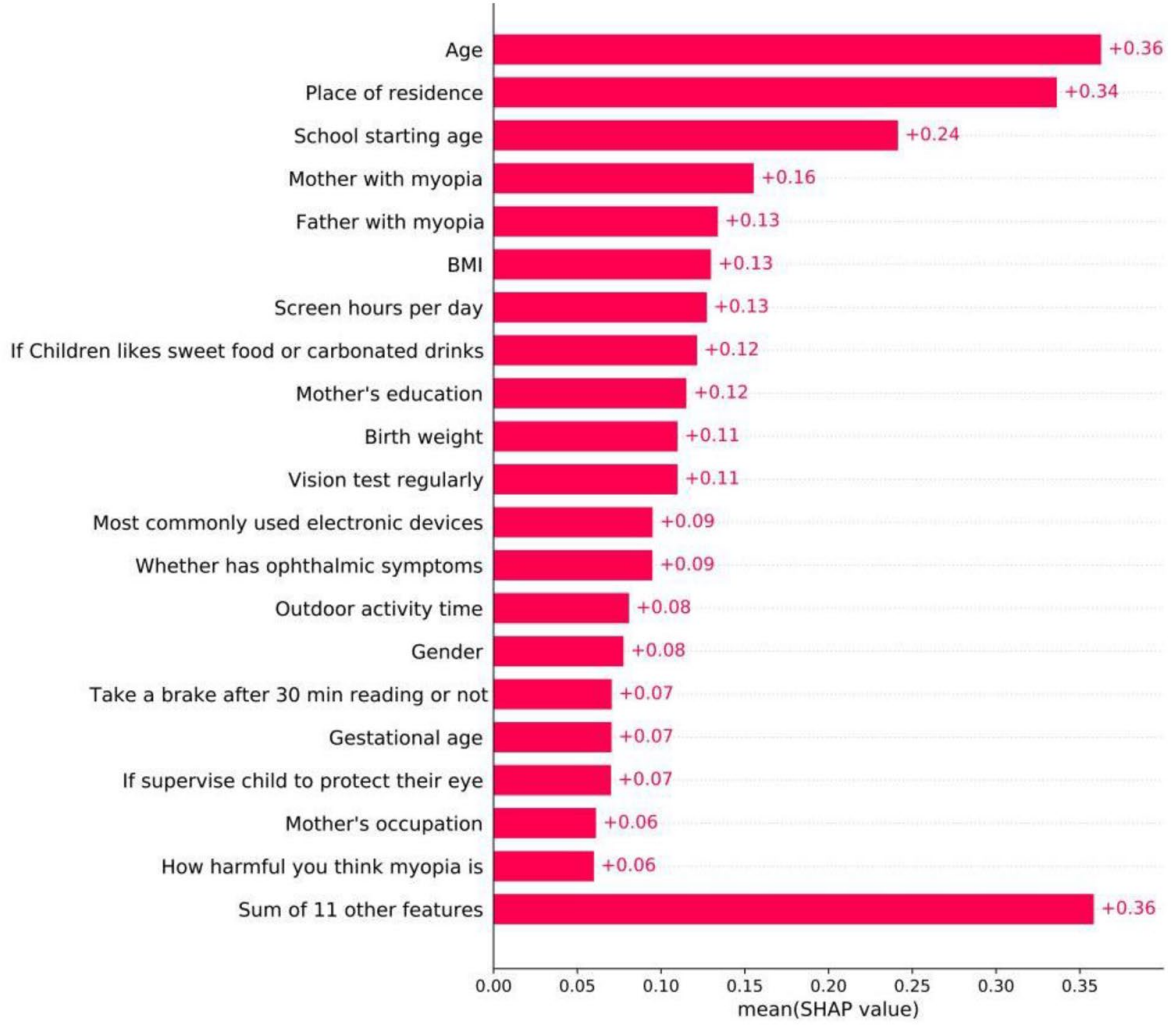
Bar chart of the average SHAP value magnitude computed from a GBDT model

In Fig. 2 of this paper, the larger the SHAP value of the influencing factor of myopia, indicating the more significant the influence of this factor on myopia when the influence of other confounding factors is reduced. The main explanation is the overall importance. As shown in Fig. 2, "Age" is the most important influencing factor in this paper. This feature alone changes the absolute probability of predicting myopia by 36% on average. Other key factors included the place of residence of elementary school students (0.34), students’ age when starting education (0.24), mother’s degree of myopia (0.16), father’s degree of myopia (0.13), student’s BMI (0.13), student’s daily electronic devices using time (0.13), student’s preference for sweets and carbonated drinks (0.12), mother's education level (0.12), student’s birth weight (0.11) and whether the student has their vision checked regularly (0.11).

**Fig. 3:**
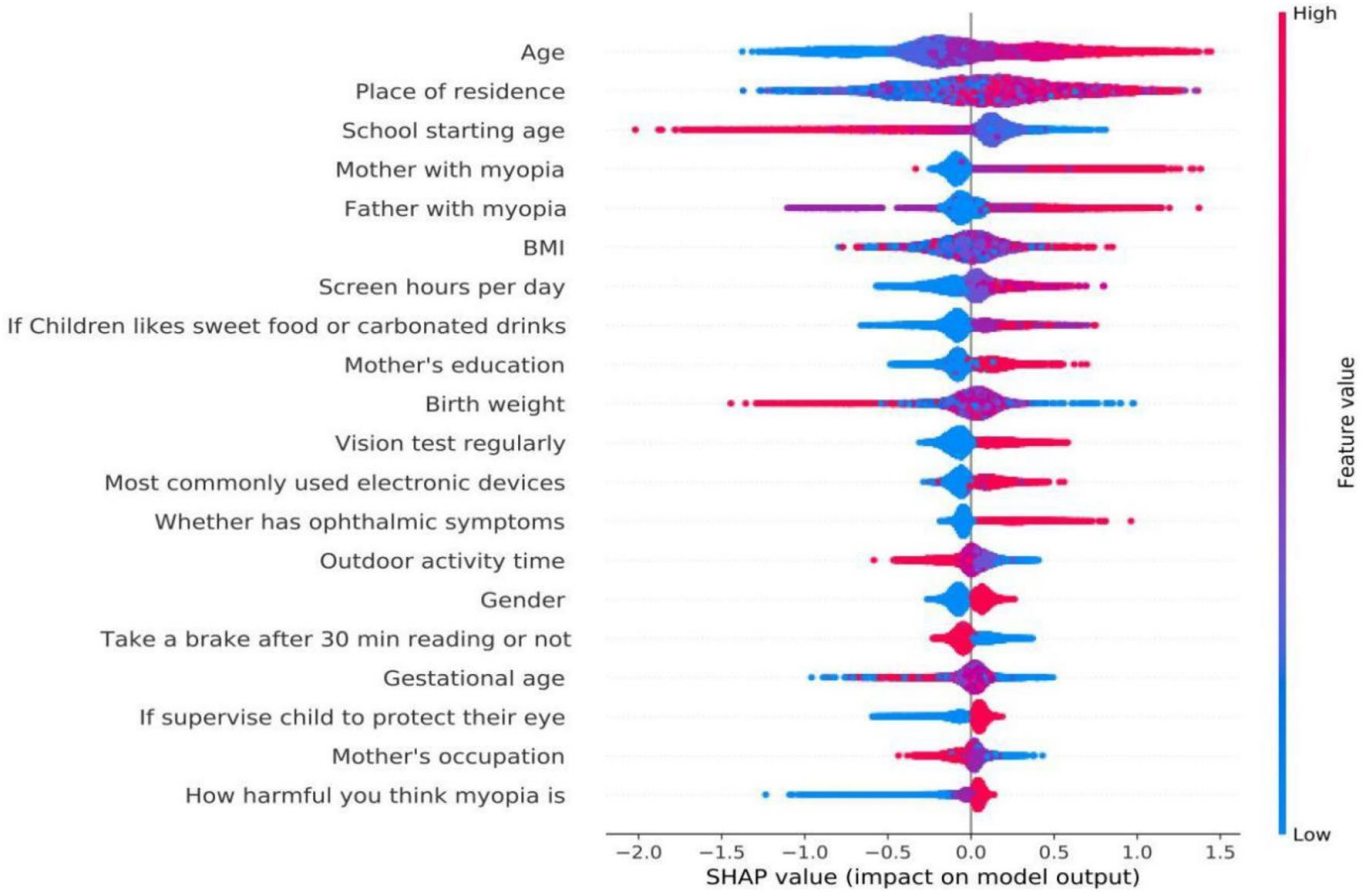
A set of beeswarm plots, where each dot corresponds to an individual subject in the study. The dot’s position on the X axis shows the positive or negative impact of each individual measurement on the prediction. The color represents the feature value (red high, blue low) for that measurement. When multiple dots land at the same X position, they pile up to show density

In Fig. 3, a point represents a sample, and all sample points are presented in the diagram. If the sample distribution is relatively scattered, the greater the influence of the feature is. The color represents the size of the feature value. The more red the color means that the value of the feature itself is larger, and the more blue the color means that the value of the feature itself is smaller. The abscissa is the size of the SHAP value, and if the SHAP value is less than 0, it means negative influence; if the SHAP value is greater than 0, it has a positive impact. For example, in the feature of "Age" in Fig. 3, the younger the age, the lower the risk of myopia, and the older the age, the higher the risk of myopia. The same analysis shows that: starting education earlier, high degree of myopia and educational level in parents, high BMI, like eating sweet food and drinking soda, higher near-work intensity, low birth weight and gestational age, and parents not bringing their children to regular vision checks are risk factors in the development of myopia. Spending prolonged periods engaging in outdoor activities are a protective factors (Fig. 3).

It is particularly interesting that BMI is predictive of myopia. According to the BMI grading standard of each age group (National Standard of People's Republic of China, WS/T 586–2018), students were divided into obesity, overweight, and other groups. The prevalence of myopia in the obese group was greater than that of the overweight group than the other groups (32.44% > 23.21% > 19.52%; *X*^2^ = 152.580, *P* < 0.001). Correlation analysis found that obese and overweight students spent longer periods of time using electronic products, reading and writing, and doing extracurricular training, and that these students preferred sweets and carbonated drinks and took less time for daily outdoor activities (*P* < 0.001).

### Analysis of the prevalence of myopia in different place of residence

**Fig. 4:**
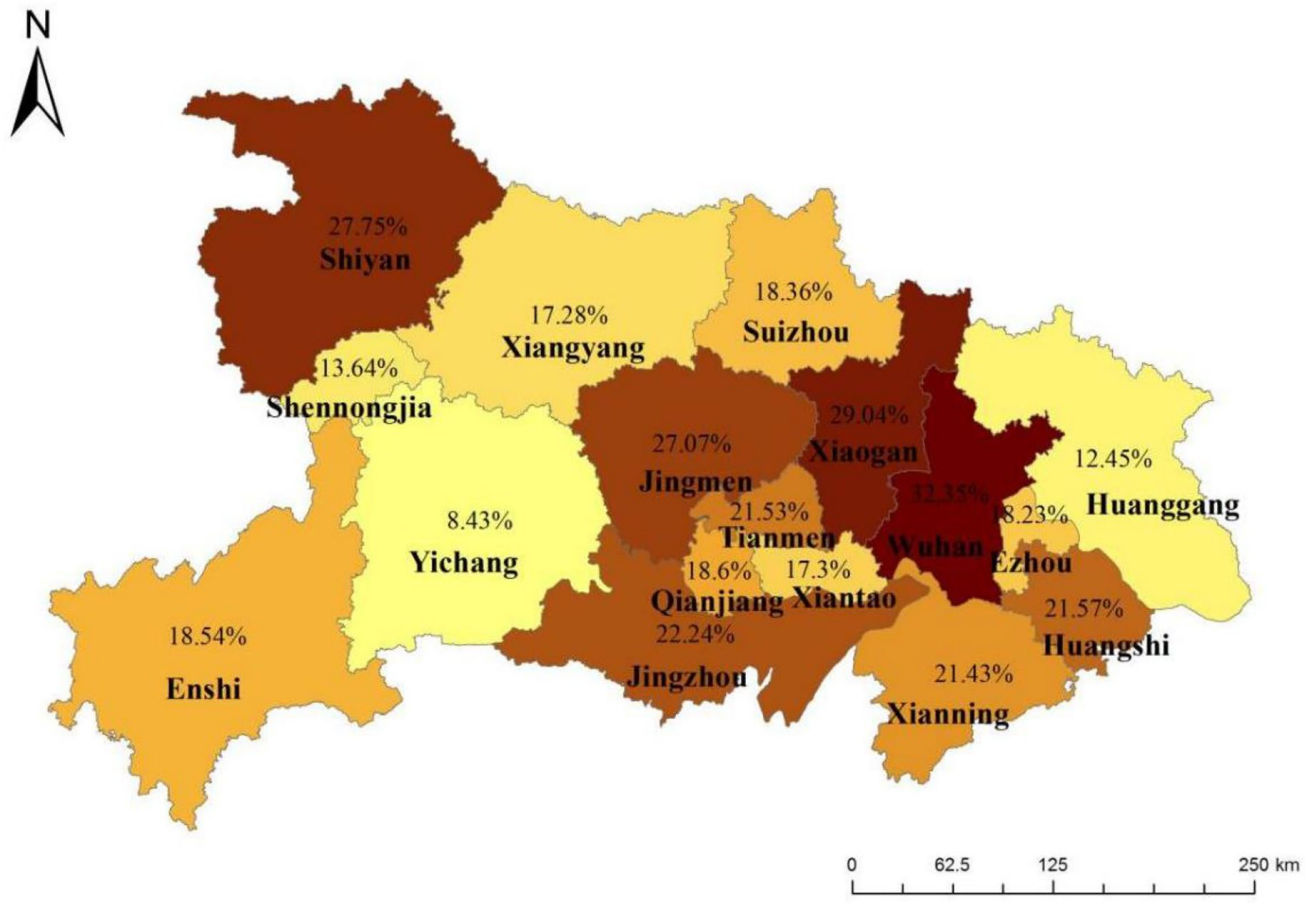
Distribution of myopia prevalence among pupils in grades 1–3 in Hubei Province

The machine-learning model found that the place of residence was an important factor predictive of myopia occurrence. The prevalence of myopia among primary school students in Wuhan (32.35%), the provincial capital city, was higher than that in other urban areas (23.03%) and rural areas (14.82%) (*χ*^2^ = 264.043, *P* < 0.001) (Fig. 4).

The prevalence of myopia and education level of parents differed across regions. Myopia prevalence in Wuhan parents was highest (father: 44.97%; mother: 39.13%), followed by other urban areas (father: 26.01%; mother: 26.23%), and the lowest in rural areas (father: 14.00%; mother: 15.46%). The proportion of fathers with higher education in provincial capital city, other urban areas and rural areas was 84.38%, 39.31% and 33.88%, respectively, while that of mothers was 82.05%, 35.33% and 27.80%, respectively (Table 4).

**Table 4:** Differences in parents of grade 1–3 students in Hubei Province between urban and rural areas

Area	Total	Father with myopia		Mother with myopia		Father's education level			Mother's education level		
		No	Yes	No	Yes	Low	Secondary	Higher	Low	Secondary	Higher
Provincial Capital	1799	55.03% (990)	44.97% (809)	60.87% (1095)	39.13% (704)	0.11% (2)	15.51% (279)	84.38% (1518)	0.11% (2)	17.84% (321)	82.05% (1476)
Urban areas	8415	73.99% (6226)	26.01% (2189)	73.77% (6208)	26.23% (2207)	2.28% (192)	58.41% (4915)	39.31% (3308)	2.84% (239)	61.83% (5203)	35.33% (2973)
Rural areas	4799	86.00% (4217)	14.00% (672)	84.54% (4057)	15.46% (742)	4.00% (192)	62.12% (2981)	33.88% (1626)	5.81% (279)	66.39% (3186)	27.80% (1334)

Degree of intensity of near works in students differed among regions. Only 48.79% of students in Wuhan spent more than 1 h on outdoor activities per day, which was less than students in other urban (57.55%) and rural areas (64.99%). On the other hand, 45.36% of students in provincial capital city spent more than 1 h in daily continuous reading and writing, a significantly larger amount than that in urban (36.36%) and rural areas (33.97%). Additionally, 31.24% of students in provincial capital city used electronic products for more than 1 h per day compared to 25.85% and 23.92% in urban and rural areas, respectively. Furthermore, 77.10%, 59.66% and 52.28% of students participated in extracurricular training across these three regions, respectively. The students in the provincial capital city spent the least time on outdoor activities, while the intensity of near works was the greatest (Table 5).

**Table 5:** Differences in eye use behavior of grade 1–3 students in Hubei Province between urban and rural areas

Area	Total	Outdoor activity time		Continuous reading and writing time		Electronic devices usage time		Extra-curricular training time	
		< 1 h	> 1 h	< 1 h	> 1 h	< 1 h	> 1 h	No	Yes
Provincial capital	1799	51.03% (918)	48.79% (881)	54.64% (983)	45.36% (816)	68.76% (1237)	31.24% (562)	22.90% (412)	77.10% (1387)
Urban areas	8415	42.45% (3572)	57.55% (4843)	63.63% (5355)	36.36% (3060)	74.15% (6240)	25.85% (2175)	40.31% (3395)	59.66% (5020)
Rural areas	4799	35.01% (1680)	64.99% (3119)	66.03% (3169)	33.97% (1630)	76.08% (3651)	23.92% (1148)	47.72% (2290)	52.28% (2509)

## Discussion

The incidence of myopia is increasing and gradually showing a trend of younger age, which has become a public health problem of global concern. Elementary school students in grades 1–3 have just started school education and are less affected by external confounding factors. The visual acuity and refractive status survey of this population can reflect the current status of myopia in children in the early education stage. Therefore, in order to evaluate the prevalence and causes of children's myopia in Hubei Province, students in grades 1–3 were selected for examination. We found that the overall prevalence of myopia in students of grades 1–3 was 21.52%, in grade 1, 2, 3 is 14.70%, 20.54% and 28.93%, respectively. Qian DJ et al. [10] carried out a large sample visual acuity survey in Yunnan Province and they also used cycloplegia for autorefraction. The myopia rate of students in the 5–8 years old group was 21.7% and was similar to our survey. Jianyong et al. [11] found that the myopia rates of primary school students in grade 1–3 were 14.8%, 32.0% and 38.4%, respectively in Yiwu County, Zhejiang Province, they did not use cycloplegia for optometry, so the prevalence of myopia was higher than in our study. This large sample screening realizes autorefraction under ciliary muscle paralysis with a large sample size, and the results can reflect the refractive state of children more truly. The data of this study can be used to interpret and compare the optometry data of cycloplegic refraction between different regions of China and other countries, to explore the possible reasons for the regional and nationality differences in the prevalence of myopia, and to seek possible measures to reduce the prevalence of myopia.

We then analyzed the influencing factors of myopia using a machine learning model to identify the most important factors correlated with myopia. We found some interesting results. First of all, we found that students' residence was an important influencing factors. The prevalence of myopia among students in grades 1–3 in Wuhan was 32.35%, significantly higher than that of other urban (23.03%) and rural students (14.82%). In order to understand the reasons for the regional differences in the prevalence of myopia, we further analyzed the influencing factors of myopia among students in different regions. We found that only 48.79% of students in Wuhan (provincial capital) spend > 1 h per day in outdoor activities, including physical education, which was significantly less than that in other urban and rural areas. In Wuhan, 45.36% of students spent > 1 h per day continuously reading and writing; 31.24% of students spend > 1 h per day on electronic products; and 77.10% of students participated in extracurricular training. These proportions in Provincial capital cities were higher than in other urban and rural areas. In addition, we found that the prevalence of myopia among parents of students in provincial capital cities is higher, and the education level of urban parents is significantly higher than that of other urban and rural areas. Parents with higher education level typically have higher academic requirements for their children, as reflected in the increased intensity of near work for Wuhan students. Therefore, we speculated that the regional differences in the prevalence of myopia among students may be related to outdoor activity time, near-work intensity and genetic factors.

In addition, this study revealed that higher BMI, a proxy for obesity, is a risk factor for myopia. Myopia prevalence in obese students was significantly higher than that of overweight students and other groups. For the interesting view that "high BMI is a risk factor for myopia" found in this study, after repeatedly consulting the relevant literature at home and abroad, we found that a survey of 728 students aged 6–7 and 898 students aged 12–13 in Ireland also found that higher BMI was related to myopia in both two age groups. It is also concluded that the prevalence of myopia in obese group is higher than that in overweight group and non-overweight group (children aged 6–7: 9.4% vs.3.5% vs.3.2%; children aged 12–13: 32.8% vs.25.8% vs.20.0%). And the significant relationship between BMI and myopia still exists after controlling relevant age, race and extracurricular sports activities [12]; Similarly, in the Netherlands, a survey of 5711 children aged 6 also found that the BMI of myopia children was higher [13]; A survey of 122 grade 3 pupils in Japan found that there was a significant positive correlation between body weight and BMI and ocular axial length [14]; A 5-year longitudinal study of 3862 kindergarten to high school students in South Korea found that the heavier the degree of myopia, the greater the average BMI (high myopia: 20.9 > moderate myopia: 20.3 > mild myopia: 19.2 > non-myopia: 17.8) [15]. Epidemiological surveys from different regions around the world have found that BMI has a certain relationship with myopia. In order to understand the reasons for the difference in the prevalence of myopia caused by BMI, we further analyzed the data and found that BMI was positively correlated with the daily use time of electronic products, continuous reading and writing time, extracurricular learning time, sweets and carbonated drinks, however negatively correlated with the time of daily outdoor activities. Therefore, we speculate that there may be two reasons for the high incidence of myopia in obese students. On the one hand, obese students lack outdoor activities and spend more time on near work, which will promote the occurrence of myopia. On the other hand, large consumption of sweets and carbonated drinks may lead to hyperglycemia and hyperinsulinemia, which will block the retinol receptor pathway, resulting in excessive proliferation of scleral tissue cells and myopia [16, 17]. However, this is only a speculation. The relationship between BMI or obesity and myopia needs further clinical and experimental research and demonstration. In the later stage, we will start clinical research on the relationship between BMI and myopia to find out the possible causes. So as to find new ideas for the prevention and treatment of myopia.

The regional differences in the prevalence of myopia and the correlation between myopia and BMI are ultimately related to the differences of genetic and environmental factors. Family studies, twin studies, DNA sequence analysis, and gene linkage analysis all show that the occurrence of myopia was related to genetic factors [18–21]. Our study confirms these findings, revealing that the prevalence of myopia in students with both myopic parents was significantly higher than that of students with one or no myopic parents. Furthermore, the higher the degree of myopia of the parents, the higher the chance to have myopia in children. Mutti et al. [22] controlled for the influence of environmental factors and found that for students with no, one, or two myopic parents, had myopia risk ratios of 1, 3.22 and 6.40, respectively, indicating that the children with two myopic parents are more likely to get myopia. In addition to genetics, we found that environmental factors imposed by parents are also the reasons for children’s proneness to myopia. For example, this study found that parents with myopia have higher levels of education. The academic requirements for children are more stringent, which makes students start to receive education at an early age, use their eyes at close range for a long time, and lack of outdoor activities. Therefore, children with myopia genetic background, especially those with high myopia parents, should pay attention to visual acuity examination from an early age.

In terms of the environment, we found that near works of all kinds was a risk factor for myopia. Low sensitivity to defocus, high accommodative hysteresis, and hyperopic defocusing of the peripheral retina under continuous close use of the eye may contribute to the development of myopia [23–25]. Wu Hao et al. [26] found that long-time near-work will reduce the opening of choroidal capillaries and blood flow, resulting in hypoxia and nutrient supply of adjacent sclera tissues. Hypoxia in sclera microenvironment activates hypoxia inducible factor-1a (HIF-1a) signal pathway, promotes fibroblast differentiation into myofibroblasts, and reduces type 1 collagen synthesis, cause scleral extracellular matrix remodeling, scleral thinning, and eventually lead to axial elongation and myopia.

In addition, we found that students who use mobile phones the most have the highest prevalence of myopia compared to those focusing on other electronic devices. We speculate that the small screen of the mobile phone, the narrow field of view, having eyes close to the screen, and the inability to guarantee regular long-distance viewing are related. By investigating these modifiable myopia risk factors, we can better identify those high-risk groups that may need intervention, so as to control the occurrence and development of myopia. Outdoor activities may have a protective effect on myopia, potentially related to the higher light levels in the outdoor environment which can causes pupil contraction, increased focus depth, reduced blurred vision, and curtailed axial growth [27]. Increased outdoor light can also promote the release of dopamine (DA), activation of D1-like receptors leads to hyperopia and that of D2-like receptors leads to myopia. Outdoor bright light can activate the dopamine D1 receptor signaling in the ON pathway in retina, make the refractive state drift farsighted, and inhibit the development of myopia [28, 29]. In addition, the animal experiments of Zhou Xuan et al. [29] found that outdoor light can delay the reduction in choroidal blood flow and choroidal thickness in myopia, so as to improve scleral hypoxia and control the progress of myopia. In future, we will conduct a clinical trial of children's outdoor activities, so as to find the threshold of light exposure intensity and duration that can protect myopia, and provide a scientific way of outdoor activities.

The incidence of myopia is related to many factors. Large-sample epidemiological surveys often have problems with large data processing workloads and complicated manual operations. The number of influencing factors included in traditional analysis methods is usually limited, resulting in low classification accuracy. Machine learning uses algorithms that are specifically geared toward finding associations between data in alternative ways to the traditional one-dimensional statistical approaches. Machine learning also takes advantage of the increasing availability of computational power and storage space to provide significantly faster outputs. As such, machine learning allows us to better utilize the increasingly complex data that is becoming available. Here, we demonstrated that the GBDT machine learning model can be better used to explore the influencing factors of myopia. We incorporated physiological characteristics, regional distribution, genetic factors, environmental factors, eating habits, and parents' perception of myopia (six major parts, 31 influencing factors in total), covering many aspects related to the incidence of myopia, and were able to use both continuous and categorical inputs without need for scaling or other pre-processing modifications. While the influencing factors may have mutual influence before, the machine learning method can reduce confounding influence among correlated factors. In addition, this algorithm was employed due to its state-of-the-art accuracy, the area under the ROC curve of the GBDT model selected in this study is 0.92, which has significantly improved performance. There were many influencing factors included in this study. Using the machine learning model, we found some influencing factors less involved in previous studies, such as students' school age, BMI, the types of electronic products most commonly used. The discovery of these influencing factors is beneficial to help formulate myopia prevention and control policies to provide some new ideas, such as strictly control the school age of primary school students, try to choose electronic products with large screen for online learning.

Overall, we suggest that students should be encouraged to enhance outdoor activities and reduce the intensity of near work to prevent the occurrence and development of myopia. However, this study also has certain limitations: as a cross-sectional clinical study, the results should to be further verified in the longitudinal study to control the important influencing factors of myopia and explore whether the occurrence and development of myopia have been improved.

## Conclusion

The prevalence rate of myopia in primary school students in Hubei Province was 21.52% and increased with grade level. Using a machine learning model, we found higher prevalence of myopia in older students, urban students, students with myopic parents, those with higher BMI, and those that engaged in greater intensities of near works. Degree of engagement in outdoor activities was negatively correlated with myopia.

## Data Availability

Please contact author for data requests.
